# Maximum heart rate and mortality in sepsis patients: a retrospective cohort study

**DOI:** 10.1007/s11739-025-03960-0

**Published:** 2025-05-13

**Authors:** Yawei Shen, Jieling Wang, Quanxia Cao, Yaohui Wu, Qingtong Wang, Nan Wang, Min Shao

**Affiliations:** 1 Department of Critical Care Medicine, The First Affiliated Hospital of Anhui Medical University, Hefei, 230032 China; 2 Anhui Public Health Clinical Center, Hefei, 230032 China; 3 Institute of Clinical Pharmacology, Anhui Medical University, Key Laboratory of Anti-inflammatory and Immune Medicine, Ministry of Education, Collaborative Innovation Center of Anti-Inflammatory and Immune Medicine, Hefei, 230032 China

**Keywords:** Maximum heart rate (MHR), Sepsis, 28-Day mortality, Intensive care unit (ICU)

## Abstract

The effects of maximum heart rate (MHR) on the prognosis of sepsis patients are still being determined, and the most optimal MHR is yet to be recommended. We aimed to determine the association between MHR and 28-day mortality in sepsis patients. A total of 269 patients with sepsis were enrolled in this retrospective cohort study. The patients were all from a 28-bed multi-disciplinary ICU in a tertiary teaching hospital. Relevant information about sepsis patients was extracted according to specific inclusion and exclusion criteria. Examined parameters such as the MHR, blood pressure, laboratory variable, primary disease, and comorbidities were collected. The study outcome was a 28-day death. A Cox proportional hazard model and restricted cubic splines (RCS) were used to elucidate the association between MHR and the 28-day mortality of sepsis patients. Sensitivity analyses were also conducted on our results. Finally, we test and verify our results using a validation cohort from a large public database. In patients with sepsis, the 28-day survivors have the lower MHR than the non-survivors (106 vs. 118, *P*<0.001). After univariate and multivariate Cox regression analyses, the MHR was significantly correlated with 28-day mortality [hazard ratio 1.013, 95% confidence interval (CI) 1.004–1.022, *P* = 0.004; hazard ratio 1.013, 95% CI 1.004-1.021, *P* = 0.004]. The patients in the highest quartile of MHR had a higher risk for 28-day mortality than other patients based on the Kaplan–Meier survival curves (log-rank *P*<0.001). This correlation remained remarkable in subgroup analysis. A typical U-shaped curve relationship was observed between MHR and the 28-day mortality as a continuous variable using the RCS model. The curve shows that either too high or too low MHR will increase the risk of death. The sepsis patients who had a MHR ranging from approximately 70 to 110 beats per minute (bpm) had the lowest 28-day mortality rate. A similar trend was identified in the validation cohort. High or low MHR was associated with an elevated risk for 28-day mortality in patients with sepsis.

**Supplementary Information:**

The online version contains supplementary material available at 10.1007/s11739-025-03960-0.

## Introduction

Sepsis is defined as life-threatening organ dysfunction caused by a dysregulated host response to infection [1]. Despite significant advancements in structured treatment strategies, it remains the leading cause of death in critically ill patients worldwide [2]. This presents a major problem for public health, with nearly 20 million cases each year and mortality rates of almost 50% for severe sepsis and 80% for septic shock [3].

A growing body of data suggests the existence of an essential heterogeneity in the type and intensity of the inflammatory status during sepsis [4]. Such heterogeneity is also manifested in heart rate, blood pressure, and other clinical signs.

Tachycardia is very common in sepsis patients. An elevated heart rate was reported to be associated with higher mortality in many diseases, such as cardiovascular disease [5], coronary artery disease [6], diabetes [7], and even cancer [8]; however, it is still not clear whether the elevated heart rate of sepsis patients is related to short-term prognosis. Besides, although the guidelines or consensus recommend the best mean arterial pressure for sepsis patients, the most suitable maximum heart rate is not recommended yet. So the association between optimum maximum heart rate and mortality risk in sepsis patients remains unknown.

Hence, to answer this question, we conducted a retrospective cohort study and sought to determine the association between maximum heart rate and 28-day mortality in sepsis patients admitted to ICU. The optimal maximum heart rate of sepsis patients was also explored.

## Methods

### Study design and participants

#### Discovery cohort

**Fig. 1:**
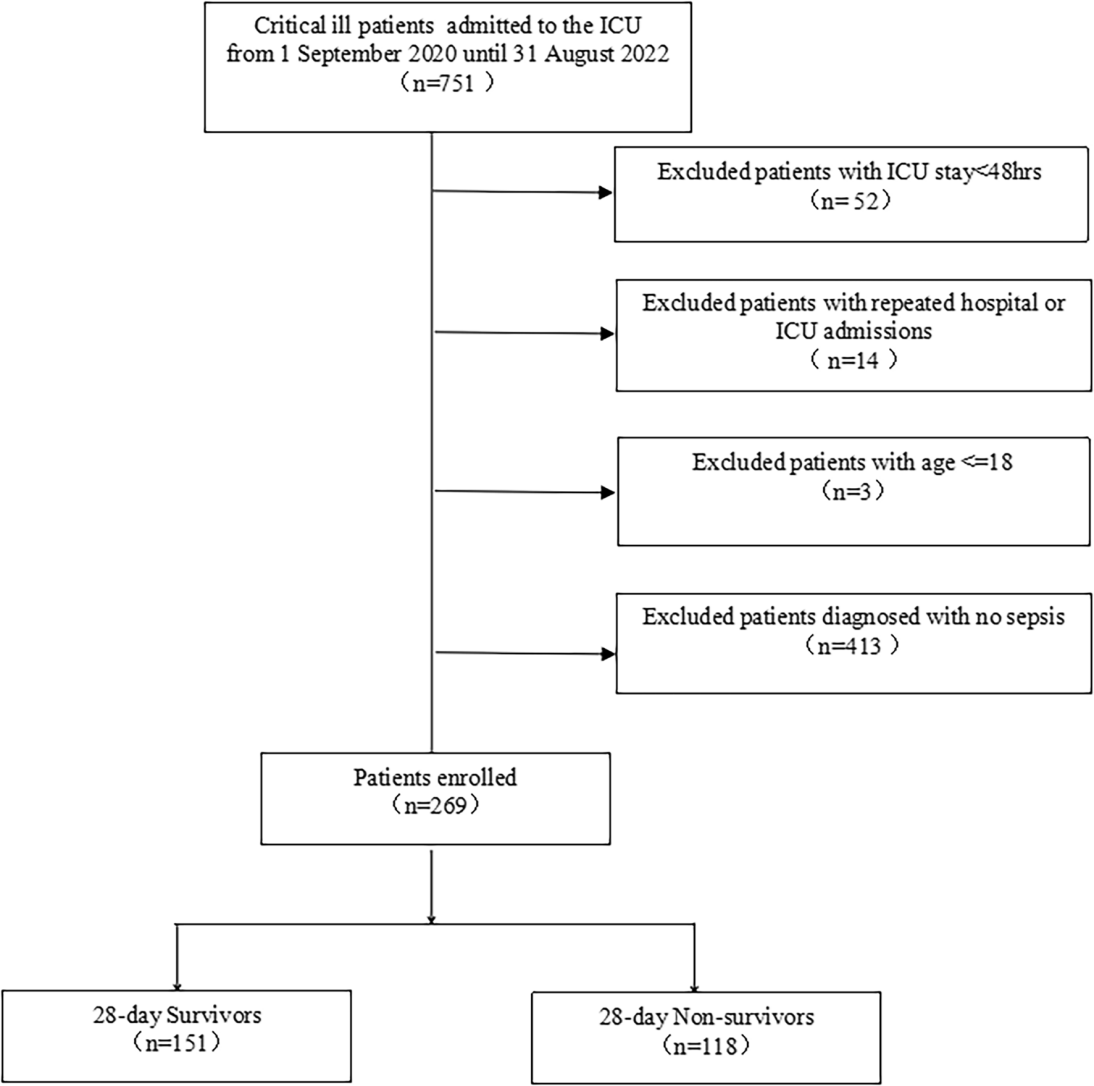
The flowchart of the study cohort selection

The discovery cohort was a single-center, retrospective study performed in a 28-bed multi-disciplinary ICU in the First Affiliated Hospital of Anhui Medical University. All medical records from 1 September 2020 until 31 August 2022 were reviewed for patients admitted to the ICU because of sepsis. According to the 2016 Surviving Sepsis Campaign (SSC) Definitions for Sepsis (Sepsis 3.0) [1], sepsis is a medical condition where organ dysfunction is caused by an infection. It is diagnosed based on a sequential organ dysfunction assessment (SOFA) score of at least two points. This includes patients who have confirmed or suspected infection. We enrolled only the first stay in the ICU of patients admitted to the ICU more than once. Patients with the following conditions were also excluded: (1) age < 18 years; (2) patients who were discharged from the ICU or died within 48 h after admission; (3)who were pregnant; and (4)who did not undergo invasive arterial blood pressure monitoring. The patient selection flow chart is shown in Fig 1. At discharge, all relevant comorbidities were identified using the diagnostic codes of ICD-9 and ICD-10. The study protocol was approved by the Medical Institutional Ethics Committee of our Hospital with a waiver of informed consent.

#### Validation cohort

The validation cohort is derived from the Medical Information Mart for Intensive Care (MIMIC)-IV database (Version 2.0) [9]. The database was developed by the Massachusetts Institute of Technology Lab for Computational Physiology as a free available public database. MIMIC-IV integrates de-identified clinical data of patients admitted to the Beth Israel Deaconess Medical Center (BIDMC, Boston, Massachusetts, United States) from 2008 to 2019. The anonymous data protects patients’ privacy so well that the requirement for informed consent is waived. One of our authors (Shen) finished the online training for the Collaborative Institutional Training Initiative program of the National Institutes of Health and had access to extract data from the MIMIC IV database (Record ID 37971581). What is more, because our study was an analysis of a publicly available database with pre-existing institutional review board (IRB) approval, our institution’s IRB approval was exempted.

Similarly, we enrolled patients who met the diagnosis of sepsis. Moreover, patients aged > 18 years old with a length of ICU stay > 48 h. Only data related to the first ICU admission were considered for patients who had ICU admission more than once. (Additional file 1: Fig S1)

### Definition of maximum heart rate

The patient’s heart rate was measured, verified, and recorded hourly, and the maximum heart rate (MHR) was defined as the patient’s highest heart rate within 24 h after ICU admission.

### Data collection

The clinical data of the eligible patients were extracted from the hospital's Electronic Medical Record (EMR). The demographics (age, sex), clinical data (maximum heart rate, blood pressure), the primary disease (skin and soft tissue infection, blood infection, lung infection, abdominal cavity infection, trauma), and the intervention strategies (norepinephrine therapy, arginine vasopressin therapy, and mechanical ventilation) were recorded in detail. Comorbidities, including coronary heart disease, diabetes, hypertension, and chronic kidney disease, were also retrieved from the EMR.

The following data were also collected: blood routine test variables included white blood cell (WBC) counts, hemoglobin (Hb), platelets (PLTs), neutrophils (NEU), lymphocytes (LYM); blood biochemical variables included alanine transaminase (ALT), total bilirubin (TB), serum creatinine (SCr), blood urea nitrogen (BUN), lactic acid (Lac). The worst value was extracted if a  laboratory variable was recorded more than once in the first 24 h after ICU admission.

We calculated Acute Physiology and Chronic Health Evaluation II (APACHE II), Sequential Organ Function Assessment (SOFA) score, and Acute Physiology score (APS) in all study patients after the first 24 h of ICU admission.

The primary outcome of this study was the 28-day mortality.

## Statistical analyses

Continuous variables were expressed as mean with SD or median with interquartile ranges, and categorical variables were reported as numbers (percentage). The baseline characteristics between different groups were analyzed using Student's *t* test or Wilcoxon rank-sum test for continuous variables. The Chi-square test was employed to compare differences in the categorical variables.

A Cox proportional hazards model was applied to determine whether MHR was independently associated with 28-day mortality after adjusting for potential confounders. Of note, covariates with statistical differences between the survival group and the non-survival group were involved in the multivariate Cox models. Potential multicollinearity was tested using a variance inflation factor, with a value > = 5 indicating multicollinearity. Kaplan–Meier survival analysis was performed for 28-day mortality, and the differences between the curves were compared using the log-rank test.

Moreover, sensitivity analysis was conducted to determine the impact of various subgroups, classified by sex, age, comorbidities, first-day APACHE score and norepinephrine usage on our results.

We also analyzed the MHR as a continuous variable using restricted cubic splines to identify potential non-linear relationships with adjusted hazard ratio. Significant discovery results were tested for replication and generalizability in validation cohort patients.

We removed samples with missing values of maximum heart rate. Covariates with more than 20% missing values were excluded. Covariates with missing values less than 20% were filled with values using multiple interpolation.

All analyses were conducted using the software Stata (version 14.0) and R (version 4.2.3, R Foundation for Statistical Computing, Vienna, Austria). Two-tailed *P*<0.05 was considered to be statistically significant.

## Results

### Discovery cohort

#### Patient characteristics and clinical features

Finally, 269 patients diagnosed with sepsis were enrolled in our study. Of all the cohort study participants, there were 151(56.13%) survivors and 118(43.87%) nonsurvivors until 28 days after ICU admission. The flowchart of the study cohort selection is shown in Fig. 1.

Table 1 shows the baseline characteristics of the participants between 28-day survivors and nonsurvivors. No significant differences were observed with respect to age or gender distribution. Compared with the survivors, the nonsurvivors had significantly higher APACHE II, SOFA, and APS scores (*P*<0.001). The nonsurvivors had significantly higher MHR but lower LSBP and LDBP than survivors. Primary diseases included skin and soft tissue infection, lung infection, and abdominal cavity infection were no significant differences. There were no differences in the prevalence of comorbidities. Moreover, we compared laboratory variables between survivors and nonsurvivors. As indicated in Table 1, the results showed that nonsurvivors had higher ALT, TB, SCr, BUN, and Lac than survivors. Additionally, nonsurvivors received more accounts for norepinephrine therapy and arginine vasopressin therapy significantly compared with the survivors.

**Table 1:** Comparisons of demographics between 28-day survivors and non-survivors

	Total (*n* = 269)	Survivors (*n* = 151)	Non-survivors (*n* = 118)	P
Male(n(%))	185 (68.77%)	105 (69.54%)	80 (67.80%)	0.76
Age(years)	67.00 (55.00, 75.00)	67.00 (54.00, 75.00)	66.00 (56.00, 76.00)	0.55
APACHEII	26.00 (21.00, 33.00)	24.00 (18.00, 28.00)	30.00 (25.00, 36.00)	<0.001
SOFA	8.00 (6.00, 12.00)	7.00 (5.00, 9.00)	10.00 (7.00, 15.00)	<0.001
APS	16.00 (12.00, 21.00)	14.00 (10.00, 18.00)	19.50 (15.00, 24.00)	<0.001
MHR(bpm)	111.00 (98.00, 125.00)	106.00 (92.00, 119.00)	118.00(103.00, 131.00)	<0.001
LSBP (mmHg)	95.00 (88.00, 105.00)	97.00 (90.00, 107.00)	91.00 (82.00, 100.00)	0.001
LDBP (mmHg)	54.00 (47.00, 60.00)	55.00 (50.00, 62.00)	52.00 (44.00, 58.00)	0.003
*Laboratory variable*				
WBC 10^9^/L	10.32 (6.17, 16.55)	9.79 (6.39, 16.00)	10.73 (5.99, 17.11)	0.51
NEU 10^9^/L	8.55 (4.79, 13.27)	8.56 (4.81, 12.74)	8.54 (4.54, 14.26)	0.95
LYM 10^9^/L	0.72 (0.40, 1.19)	0.66 (0.40, 1.16)	0.84 (0.36, 1.40)	0.18
Hb g/dL	101.00 (79.00, 125.00)	107.00 (84.00, 125.00)	92.00 (70.00, 125.00)	0.024
PLT 10^9^/L	155.50 (86.50, 235.00)	169.00 (118.00,237.00)	128.00 (48.00, 208.00)	0.001
ALT u/L	36.75 (21.00, 72.00)	32.00 (20.00, 53.00)	41.00 (21.50, 122.50)	0.018
TB	17.80 (10.90, 31.30)	16.75 (10.70, 25.60)	23.60 (11.00, 42.70)	0.007
SCr umol/L	90.50 (54.90, 174.50)	79.00 (49.60, 123.00)	113.00 (64.80, 228.20)	<0.001
BUN	11.00 (7.10, 17.90)	9.40 (6.10, 15.30)	14.00 (8.30, 20.06)	<0.001
Lac mmol/L	2.60 (1.50, 5.80)	1.90 (1.30, 3.30)	4.20 (2.40, 10.30)	<0.001
*Primary disease*				
Skin and soft tissue infection (n(%))	37 (13.75%)	25 (16.56%)	12 (10.17%)	0.13
Blood infection (n(%))	35 (13.01%)	12 (7.95%)	23 (19.49%)	0.005
Lung infection (n(%))	175 (65.06%)	93 (61.59%)	82 (69.49%)	0.18
Abdominal cavity infection (n(%))	94 (34.94%)	57 (37.75%)	37 (31.36%)	0.28
Trauma (n(%))	26 (9.67%)	20 (13.25%)	6 (5.08%)	0.025
*Comorbidities*				
CHD (n(%))	35 (13.01%)	19 (12.58%)	16 (13.56%)	0.81
Diabetes (n(%))	57 (21.19%)	32 (21.19%)	25 (21.19%)	1.00
Hypertension (n(%))	87 (32.34%)	49 (32.45%)	38 (32.20%)	0.97
CKD (n(%))	41 (15.24%)	19 (12.58%)	22 (18.64%)	0.17
*Intervention strategies*				
NE (n(%))	151 (56.13%)	74 (49.01%)	77 (65.25%)	0.008
AVP (n(%))	37 (13.75%)	9 (5.96%)	28 (23.73%)	<0.001
MV (n(%))	163 (60.59%)	91 (60.26%)	72 (61.02%)	0.90

*APACHE II* acute physiologic and chronic health evaluation II, *SOFA* sequential organ failure assessment score, *APS* acute physiology score, *MHR* maximum heart rate, *LSBP* the 1 st day lowest systolic blood pressure, *LDBP* the 1 st day lowest diastolic blood pressure, *WBC* white blood cell, *NEU* neutrophil, *LYM* lymphocyte, *Hb* hemoglobin, *PLT* platelet, *ALT* alanine transaminase, *TB* total bilirubin, *SCr* serum creatinine, *BUN* blood urea nitrogen, *Lac* lactic acid, *CHD* coronary heart disease, *CKD* chronic kidney disease, *NE* norepinephrine, *AVP* arginine vasopressin, *MV* mechanical ventilation

#### Association of MHR with 28-day mortality

We further evaluated the association of MHR as a continuous variable with 28-day mortality using the Cox proportional hazards model. The results are shown in Table 2. In unadjusted analysis, Each one beat per minute (bpm) increase in MHR was associated with a 1.3% higher hazard ratio of 28-day mortality (HR 1.013, 95%CI 1.004–1.022, *P* = 0.004). Model 1, adjusted for age, indicated that each one bpm increase in MHR was associated with a 1.4% higher hazard ratio of 28-day mortality (HR 1.014, 95%CI 1.005–1.023, *P* = 0.002). This relationship remained highly significant after a series of covariates adjustment in models 2, 3, and 4. Model 5 was further adjusted for 1 st day lowest systolic blood pressure, 1 st day lowest diastolic blood pressure, and norepinephrine usage. Similarly, results indicated that each bpm increase in MHR was associated with a 1.3% higher hazard ratio of 28-day mortality (HR 1.013, 95%CI 1.004–1.021, *P* = 0.004).

**Table 2:** Association between MHR and 28-day mortality of sepsis patients

28-day mortality			
	Hazard ratio	95%CI	*P* value
Unadjusted	1.013	(1.004–1.022)	0.004
Model1	1.014	(1.005–1.023)	0.002
Model2	1.015	(1.006–1.024)	0.001
Model3	1.014	(1.005–1.023)	0.002
Model4	1.013	(1.005–1.022)	0.002
Model5	1.013	(1.004–1.021)	0.004

Hazard ratio and 95% CI for MHR in 28-day mortality were calculated using different Cox regression models. Model 1 adjusted for age. Model 2 adjusted for model 1 plus Hb and PLT. Model 3 adjusted for model 2 plus ALT and TB. Model 4 adjusted for model 3 plus SCr and Lac Model 5 adjusted for model 4 plus LSBP, LDBP and NE

#### Association between different MHR groups and 28-day mortality

**Fig. 2:**
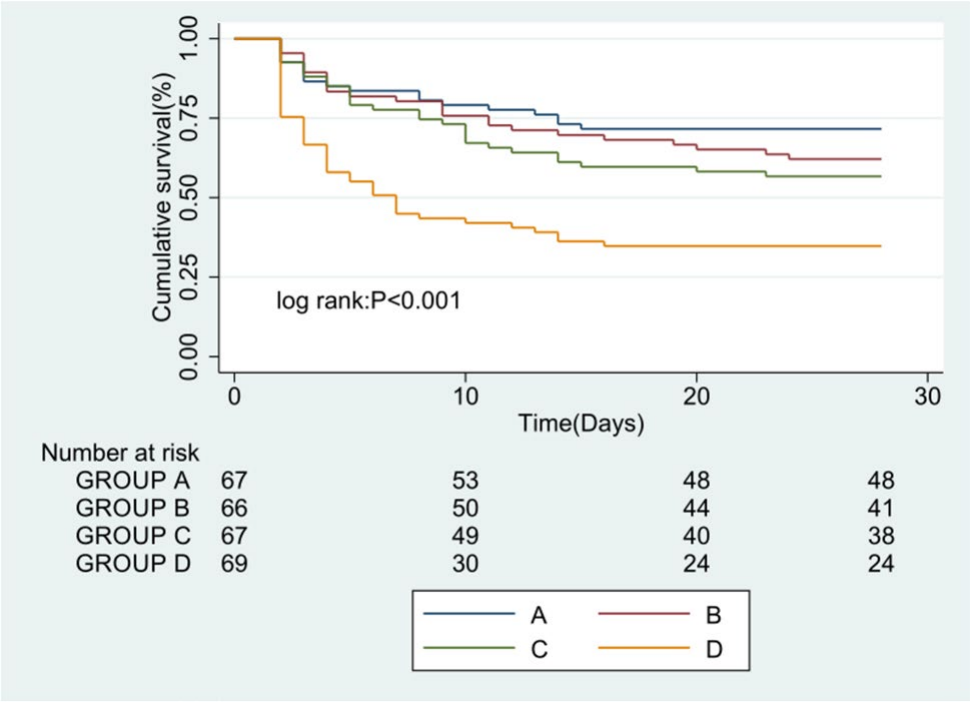
Kaplan–Meier curves of 28-day mortality by MHR

The Kaplan–Meier survival curves revealed the correlation between MHR as a categorical variable and study outcome (Fig. 2). We divided the patients into four groups according to the quartile of MHR. GROUP A was the patients who had MHR no more than 98 bpm, GROUP B was the patients who had MHR between 98 and 111 bpm, GROUP C was the patients who had MHR between 111 and 125 bpm, while GROUP D was the patients who had MHR more than 125 bpm. The Kaplan–Meier curves show that GROUP D has a lowest 28-day probability of survival than other groups (log-rank: *P* <0.001).

#### Sensitivity analyses

To further clarify the influence of MHR on the short-term prognosis of sepsis patients, sensitivity analyses were conducted based on sex, age levels, coronary heart disease, diabetes, hypertension, chronic kidney disease, APACHE II score and norepinephrine usage (Table 3). GROUP A acts as the reference group. The hazard ratio for GROUP D was more than 1 in the discovery cohort (*P*<0.05). It suggested that the 28-day mortality significantly increased in the highest quartile after full adjustments. The same trend was observed in all of the subgroups except the subgroup of female sex (HR 2.225, 95%CI 0.763–6.486, *P* = 0.143), the subgroup with age more than 70 (HR 1.997, 95%CI 0.874–4.562, *P* = 0.101), the subgroup with CHD (HR 2.679, 95%CI 0.556–12.907, *P* = 0.219) and the subgroup with CKD (HR 2.887, 95%CI 0.377–22.125, *P* = 0.308). Nevertheless, for GROUP B and GROUP C, there was no significant difference in hazard ratios compared to GROUP A.

**Table 3:** Association between MHR group and 28-day mortality of sepsis patients in different subgroups

28-day mortality									
			MHR< = 98 GROUP A	98<MHR< = 111 GROUP B		111<MHR< = 125 GROUP C		MHR>125 GROUP D	
Subgroup		N	Hazard ratio	Hazard ratio 95%CI	*P* value	Hazard ratio 95%CI	*P* value	Hazard ratio 95%CI	*P* value
TOTAL		269	Ref.	1.095(0.594–2.017)	0.771	1.580(1.862–2.895)	0.139	2.741(1.551–4.845)	0.001
Sex	Male	185	Ref.	1.282(0.608–2.701)	0.514	2.128(1.022–4.428)	0.044	3.038(1.514–6.097)	0.002
	Female	84	Ref.	0.526(0.154–1.799)	0.306	0.413(0.111–1.530)	0.185	2.225(0.763–6.486)	0.143
Age	> = 70	114	Ref.	0.958(0.395–2.324)	0.925	1.645(0.628–4.308)	0.311	1.997(0.874–4.562)	0.101
	<70	155	Ref.	1.306(0.518–3.289)	0.572	1.840(0.755–4.483)	0.180	3.995(1.657–9.631)	0.002
CHD	Yes	35	Ref.	2.232(0.262–18.982)	0.462	8.73e-19(0–/)	1.000	2.679(0.556–12.907)	0.219
	No	234	Ref.	1.155(0.585–2.279)	0.679	1.678(0.859–3.278)	0.130	2.932(1.524–5.643)	0.001
Diabetes	Yes	57	Ref.	0.722(0.169–3.075)	0.659	1.247(0.305–5.107)	0.759	3.576(1.003–12.743)	0.049
	No	212	Ref.	1.085(0.526–2.237)	0.825	1.737(0.851–3.546)	0.129	3.306(1.672–6.534)	0.001
Hypertension	Yes	87	Ref.	0.616(0.191–1.987)	0.418	1.294(0.417–4.018)	0.655	3.637(1.306–10.134)	0.014
	No	182	Ref.	1.296(0.598–2.808)	0.511	1.703(0.790–3.672)	0.174	2.995(1.439–6.236)	0.003
CKD	Yes	41	Ref.	0.639(0.108–3.795)	0.622	1.433(0.243–8.445)	0.691	2.887(0.377–22.125)	0.308
	No	228	Ref.	1.309(0.644–2.661)	0.457	1.802(0.892–3.639)	0.101	3.476(1.826–6.620)	<0.001
APACHEII	> = 25	162	Ref.	0.862(0.418–1.776)	0.687	1.032(0.488–2.185)	0.934	2.156(1.105–4.207)	0.024
	<25	107	Ref.	1.857(0.508–6.788)	0.349	3.768(1.225–11.594)	0.021	3.875(1.101–13.638)	0.035
NE	Yes	151	Ref.	0.879(0.409–1.890)	0.742	1.208(0.557–2.619)	0.633	2.609(1.317–5.169)	0.006
	No	118	Ref.	1.838(0.611–5.526)	0.278	2.770(0.940–8.166)	0.065	3.522(1.154–10.747)	0.027

Hazard ratios of 28-day mortality risk on the stratification of sex, age levels, coronary heart disease, diabetes, hypertension, chronic kidney disease and APACHEII. Adjusted variables included age, Hb, PLT, ALT, TB, SCr, and Lac. *CI* confidence interval, *Hb* hemoglobin, *PLT* platelet, *ALT* alanine transaminase, *TB* total bilirubin, *SCr* serum creatinine, *Lac* lactic acid

#### Non-linear association between MHR and outcome

**Fig. 3:**
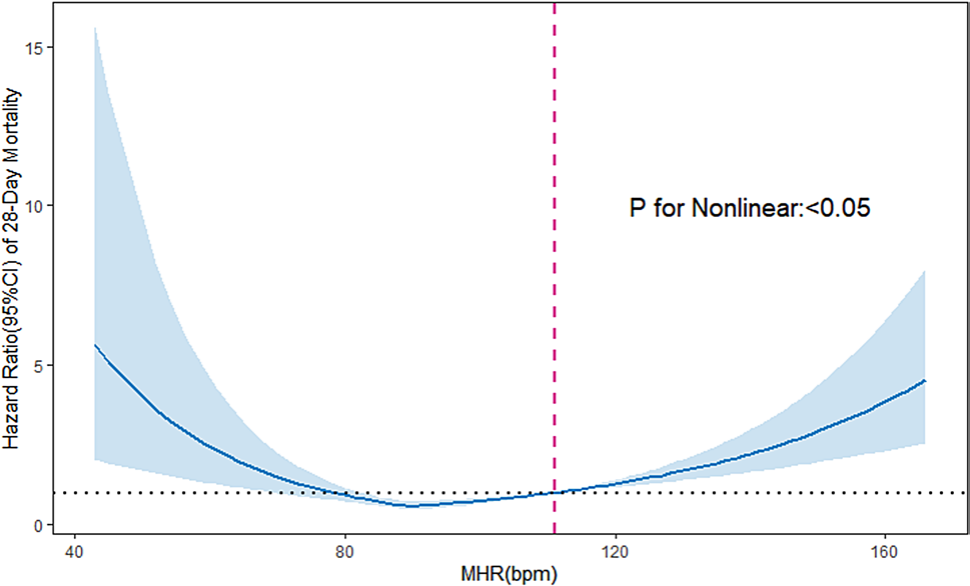
Association between MHR and hazard ratio(95% CI) of 28-day mortality using a restricted cubic spline regression model. Adjusted variables included age, Hb, PLT, ALT, TB, SCr, Lac, LSBP, LDBP, and NE. The reference (hazard ratio = 1, horizontal dotted line) was an MHR of 112 bpm (vertical dotted line). Solid lines indicate *HRs*, and shadow shape indicate 95% CIs. HRs hazard ratios, CI confidence interval

Using restricted cubic spline analysis, we observed an apparent nonlinear relationship between MHR and 28-day mortality of sepsis patients. In models with full adjustment variables, the relationship between MHR and study outcome can be described as a typical U-shaped curve (Fig. 3). The results showed that the 28-day mortality significantly increased when MHR at both ends of the curve. It indicated that either too high or too low MHR will increase the risk of death. Based on the curve, it showed that the patients had the lowest mortality at MHR of approximately 70–110 bpm.

### Validation cohort

For the validation cohort, we assessed the relationship between MHR and study outcome in sepsis patients from the MIMIC IV database (Additional file 1: Fig S1). Due to the large number of observations, we entered more covariates into the Cox proportional hazards regression model (Additional file 1: Table S2). It was found that MHR was still significantly correlated with 28-day mortality using univariate and multivariate Cox analysis. Furthermore, we divided the patients into four groups according to the quartile of MHR. A similar trend was observed that the highest quartile of MHR has the most increased 28-day mortality using Kaplan–Meier survival curves analysis (log-rank: *P*<0.001) (Additional file 1: Fig S2).

GROUP 1 (MHR< = 92) was used as a reference and hazard ratios were reported for other groups. For GROUP 4 (MHR>121), the correlation between MHR and outcome was still statistically significant in sepsis patients from different subgroups for the full model (Additional file 1: Table S3). The hazard ratios of 28-day mortality were still significantly increased in most subgroups except the subgroup with CHD (HR 1.337, 95%CI 0.953–1.875, *P* = 0.093), the subgroup with APACHE III > = 50 (HR 1.136, 95%CI 0.996–1.294, *P* = 0.057), and the subgroup with APACHE III <50 (HR 1.095, 95%CI 0.731–1.639, *P* = 0.660). Our findings were still robust in the subgroup analysis.

Similarly, MHR and outcomes of sepsis patients were found to have an apparent nonlinear relationship when used RCS analysis (Additional file 1: Fig S3). The relationship could be described as a ‘U’ type curve when examining MHR as a continuous variable.

## Discussion

In this retrospective cohort study, we analyzed the clinical data of 269 patients diagnosed with sepsis in a 28-bed multi-disciplinary ICU in the tertiary teaching hospital. And we found that the 28-day mortality risk of sepsis patients increased with the increase of maximum heart rate within 24 h after ICU admission. Then we further divided the patients into four groups according to the quartile of MHR. We observed that the patients in the highest quartile of MHR had a higher risk for 28-day mortality than other patients.

Sepsis patients admitted to ICU often have severe hemodynamic disorders manifested as significant abnormalities in blood pressure and heart rate. The heart is one of many organs affected by sepsis. On the one hand, the heart is usually in the hyperkinetic circulatory state and has supranormal ejection fraction in sepsis patients, which is a compensatory performance. On the other hand, 10%–70% of sepsis patients have sepsis-induced myocardial dysfunction, this includes not only depression of systolic function, but also diastolic dysfunction, and right ventricular dysfunction. In addition, the application of vasoactive drugs can act on the cardiovascular system and lead to abnormal heart rate. So heart rate results from the combined effects of disease severity, systemic inflammatory response intensity, and response to treatment.

Studies have demonstrated that heart rate is a risk factor in predicting prognosis in patients with myocardial infarction (MI) [10], acute type A aortic dissection [11], type 2 diabetes [12], acute ischemic stroke [13], acute intracerebral hemorrhage [14], and even in healthy individuals [15].

Although the heart rate response to sepsis may be an adaptive reaction to maintain oxygen delivery, elevated heart rate was also reported as an independent risk factor for mortality during sepsis [16]. Until now, there is no recognized heart rate as a control target in early sepsis, so we chose the first-day MHR as the study variable.

Xiao found that the cumulative and interactive effects of exposure to low diastolic arterial pressure (< = 40 mmHg) and tachycardia (> = 100 bpm) were associated with an increased risk of death. The optimal ranges for heart rate in patients with septic shock are 60–90 bpm [17]. Our study demonstrated that the MHR is a risk factor to predict 28-day mortality even after adjusting for clinical confounders. Besides, considering that many patients with sepsis will require vasopressor support to maintain circulation perfusion. Vasopressor agents can predispose patients to tachycardia and arrhythmias, which increases the risk of adverse cardiovascular events [18]. We performed a subgroup analysis, which revealed that the 28-day mortality was significantly increased in the patients with the highest quartile of MHR whether or not to use norepinephrine. Moreover, the relationship remained stable regardless of gender, age, comorbidities, and APACHE II score.

This may be due to significant tachycardia can impair left ventricular diastolic filling (with a reduction in stroke volume), compromise coronary blood flow, and increase myocardial oxygen demand. Moreover, sustained high activation levels can be harmful and lead to sympathetic autonomic dysfunction syndrome. This occurs in the early stages of sepsis. The intensity is associated with greater severity of clinical symptoms and mortality, which reflect adrenergic sympathetic overactivation [19].

Additionally, we used the RCS model to assess whether there is a curvilinear association between MHR and 28-day mortality in sepsis. After adjusting for potential confounders, a non-linear relationship was observed between MHR and 28-day mortality. According to the RCS curve, the risk of 28-day death was increased with increasing MHR when MHR exceeded approximately 110. And the risk of 28-day mortality was also significantly increased with decreasing MHR when MHR was below approximately 70.

It revealed that bradycardia is also associated with poor short-term outcomes. The harm of bradycardia has been shown in the study of many diseases. Luo [20] found through retrospective analysis that low heart rate is a risk factor for mortality, and the patients with low minimum heart rate had poor prognosis after cardiac surgery. In addition, bradycardia during gas insufflation appears to be a critical early warning sign for possible impending and unexpected cardiac arrest during routine laparoscopic surgery [21]. Another study pointed out that intraoperative bradycardia events could significantly affect outcomes when hemodynamic perturbations occur [22]. To the best of our knowledge, the presence of atrial fibrillation among sepsis-related hospitalizations is a marker of poor prognosis and increased mortality [23, 24]. Several RCT studies have found that bradycardia may be linked to increased atrial fibrillation under certain circumstances [25–27]. This because bradycardia can cause dispersion of atrial repolarization which can initiate atrial fibrillation [28].

The previous report provides strong evidence for the association between bradycardia and the outcome of critical illness. There are few literature reports on the short-term prognosis of sepsis patients with bradycardia. Now our study found that the low MHR in sepsis patients is also significantly related to poor prognosis. This means the sepsis patients had a higher risk for 28-day mortality when the MHR was too low or too high. The first-day MHR between 70 and 110 bpm in sepsis patients may benefit prognosis. The results also applied to the validation cohort.

## Limitations

First, owing to the nature of retrospective research, selection bias could not be excluded. On the other hand, we exclude patients who discharged or died within 48 h of admission to avoid any interference factors due to incomplete data or incomplete treatment. This maybe another source of selection bias. But the study provided a meaningful perspective for further research to establish a definitive causal link.

Second, the incidence of bradycardia is relatively small, which leads to data imbalance. Although every effort has been made to adjust for the confounding factors using multivariate analysis and external validation, larger sample size and multicenter cohort studies are needed to explore further.

Third, no correlation between MHR and 28-day mortality was observed in some subgroups in discovery cohort and validation cohort. This may be indicated that the predictive value of MHR varies in different populations. Consequently, prospective and multicenter cohort studies are needed to explore further.

## Conclusions

In aggregate, by analyzing a retrospective cohort and a validation cohort, our study shows that both tachycardia and bradycardia are associated with elevated risk for 28-day mortality but in a complex form in patients with sepsis. These findings suggest the potential of MHR as an early risk indicator for short-term prognosis in patients with sepsis and a potential therapeutic target, but further validation is needed through RCT studies.

## Supplementary Information

Below is the link to the electronic supplementary material.

Supplementary file1 (DOCX 125 kb)

## Data Availability

Data are available from the author YS and MS upon reasonable request.

## Abbreviations

ICU: Intensive care unit
MIMIC IV: Medical information mart for intensive care IV
ICD: International classification of diseases
APACHEII: Acute physiologic and chronic health evaluation II
APACHE III: Acute Physiology and chronic health evaluation III
SOFA: Sequential organ failure assessment score
APS: Acute physiology score
MHR: Maximum heart rate
LSBP: The 1^st^ day lowest systolic blood pressure
LDBP: The 1^st^ day lowest diastolic blood pressure
WBC: White blood cell
NEU: Neutrophil
LYM: Lymphocyte
Hb: Hemoglobin
PLT: Platelet
ALT: Alanine transaminase
TB: Total bilirubin
SCr: Serum creatinine
BUN: Blood urea nitrogen
Lac: Lactic acid
CHD: Coronary heart disease
CKD: Chronic kidney disease
NE: Norepinephrine
AVP: Arginine vasopressin
MV: Mechanical ventilation
CI: Confidence interval
HR: Hazard ratio
