# Supplementary material for: Maximum heart rate and mortality in sepsis patients: a retrospective cohort study
Source: Intern Emerg Med. 2025 May 13;21(2):621–30. doi: 10.1007/s11739-025-03960-0 (PMC13061839; doi:10.1007/s11739-025-03960-0)
Supplement: Supplementary file 1 — Supplementary file1 (DOCX 125 kb) [file 11739_2025_3960_MOESM1_ESM.docx]

**Maximum heart rate and mortality in sepsis patients: a retrospective cohort study**

Yawei Shen ^12^, Jieling Wang^12^, Quanxia Cao^12^, Yaohui Wu^12^, Qingtong Wang^3^, Nan Wang^12^* and Min Shao^1^*

1.Department of Critical Care Medicine, The First Affiliated Hospital of Anhui Medical University, Hefei 230032,China.

2.Anhui Public Health Clinical Center, Hefei 230032,China.

3.Institute of Clinical Pharmacology, Anhui Medical University; Key Laboratory of Anti-inflammatory and Immune Medicine, Ministry of Education; Collaborative Innovation Center of Anti-Inflammatory and Immune Medicine, Hefei 230032, China

* Correspondence to:

Nan Wang

E-mail: [wangnan@ahmu.edu.cn](mailto:wangnan@ahmu.edu.cn)

Min Shao

1. mail: shaominAHMU@outlook.com

**Additional file 1**

**Table S1 Comparisons of demographics between 28-day survivors and non-survivors**

|  | Total  (n = 11275) | Survivors  (n =9072) | Non-survivors  (n = 2203) | P |
| --- | --- | --- | --- | --- |
| Male(n(%)) | 6581 (58.37%) | 5343 (58.90%) | 1238 (56.20%) | 0.021 |
| Age(years) | 67.41 (55.93, 78.08) | 66.27 (54.84, 76.85) | 72.80 (60.92, 82.51) | <0.001 |
| APACHEIII | 58.00 (41.00, 78.00) | 53.00 (38.50, 72.00) | 79.00 (61.00, 100.00) | <0.001 |
| SOFA | 7.00 (5.00, 10.00) | 7.00 (5.00, 10.00) | 9.00 (7.00, 13.00) | <0.001 |
| APS | 41.00 (33.00, 51.00) | 39.00 (31.00, 49.00) | 49.00 (40.00, 59.00) | <0.001 |
| MHR(bpm) | 106.00 (92.00, 121.00) | 105.00 (92.00, 120.00) | 109.00 (94.00, 125.00) | <0.001 |
| LSBP (mmHg) | 86.00 (77.00, 95.00) | 86.00 (78.00, 95.00) | 84.00 (75.00, 93.00) | <0.001 |
| LDBP (mmHg) | 44.00 (38.00, 50.00) | 44.00 (39.00, 50.00) | 43.00 (36.00, 50.00) | <0.001 |
| **Laboratory variable** | | | | |
| WBC 10^9^/L | 14.90 (10.80, 20.00) | 14.80 (10.80, 19.80) | 15.30 (10.80, 21.00) | 0.010 |
| NEU 10^9^/L | 10.44 (8.88, 13.06) | 10.44 (8.85, 12.54) | 10.44 (9.10, 14.68) | <0.001 |
| LYM 10^9^/L | 1.43 (0.75, 1.43) | 1.43 (0.80, 1.43) | 1.12 (0.56, 1.43) | <0.001 |
| Hb g/dL | 9.60 (8.20, 11.20) | 9.60 (8.20, 11.20) | 9.50 (8.00, 11.20) | 0.11 |
| PLT 10^9^/L | 155.00 (106.00, 219.00) | 154.00 (108.00, 216.00) | 158.00 (97.00,  230.00) | 0.73 |
| ALT u/L | 103.00 (26.00, 234.71) | 140.00 (26.00, 234.71) | 57.00 (23.00, 234.71) | <0.001 |
| TB mg/dL | 2.10 (0.60, 2.24) | 2.24 (0.60, 2.24) | 1.30 (0.60, 2.24) | 0.025 |
| SCr mg/dL | 1.20 (0.90, 1.90) | 1.10 (0.80, 1.80) | 1.50 (1.00, 2.40) | <0.001 |
| BUN mmol/L | 24.00 (16.00, 39.00) | 22.00 (15.00, 35.00) | 33.00 (21.00, 53.00) | <0.001 |
| Lac mmol/L | 2.30 (1.50, 3.80) | 2.30 (1.50, 3.60) | 2.60 (1.60, 4.60) | <0.001 |
| PaCO_2_ mmHg | 34.00 (30.00, 39.00) | 35.00 (30.00, 39.00) | 34.00 (29.00, 40.00) | <0.001 |
| Bicarbonate mmol/L | 21.00 (18.00, 23.00) | 21.00 (18.00, 24.00) | 19.00 (16.00, 23.00) | <0.001 |
| **Primary disease** | | | | |
| Lung infection (n(%)) | 6788 (60.20%) | 5129 (56.54%) | 1659 (75.31%) | <0.001 |
| Abdominal cavity infection (n(%)) | 3537 (31.37%) | 2793 (30.79%) | 744 (33.77%) | 0.007 |
| Urinary infection (n(%)) | 2794 (24.78%) | 2255 (24.86%) | 539 (24.47%) | 0.70 |
| Bloodstream infection (n(%)) | 2193 (19.45%) | 1560 (17.20%) | 633 (28.73%) | <0.001 |
| **Comorbidities** | | | | |
| CHD (n(%)) | 1350 (11.97%) | 1067 (11.76%) | 283 (12.85%) | 0.16 |
| Diabetes (n(%)) | 3435 (30.47%) | 2771 (30.54%) | 664 (30.14%) | 0.71 |
| Hypertension (n(%)) | 7270 (64.48%) | 5835 (64.32%) | 1435 (65.14%) | 0.47 |
| CKD (n(%)) | 2440 (21.64%) | 1855 (20.45%) | 585 (26.55%) | <0.001 |
| **Medical Management** | | | | |
| NE (n(%)) | 4801 (42.58%) | 3394 (37.41%) | 1407 (63.87%) | <0.001 |
| AVP (n(%)) | 1796 (15.93%) | 1086 (11.97%) | 710 (32.23%) | <0.001 |
| MV (n(%)) | 10654 (94.49%) | 8639 (95.23%) | 2015 (91.47%) | <0.001 |

Abbreviations: APACHE III acute physiologic and chronic health evaluation III, SOFA sequential organ failure assessment score, APS acute physiology score, MHR maximum heart rate, LSBP the 1 st day lowest systolic blood pressure, LDBP the 1 st day lowest diastolic blood pressure, WBC white blood cell, NEU neutrophil, LYM lymphocyte,Hb hemoglobin, PLT platelet, ALT alanine transaminase, TB total bilirubin, SCr serum creatinine, BUN blood urea nitrogen, Lac lactic acid, CHD coronary heart disease, CKD chronic kidney disease, NE norepinephrine, AVP arginine vasopressin, MV mechanical ventilation.

**Table S2 Association between MHR and 28-day mortality of sepsis patients**

| 28-day mortality | | | |
| --- | --- | --- | --- |
|  | Hazard ratio | 95%CI | P value |
| unadjusted | 1.006 | (1.004-1.008) | <0.001 |
| Model1 | 1.010 | (1.008-1.012) | <0.001 |
| Model2 | 1.007 | (1.005-1.009) | <0.001 |
| Model3 | 1.007 | (1.005-1.009) | <0.001 |
| Model4 | 1.007 | (1.005-1.009) | <0.001 |
| Model5 | 1.006 | (1.004-1.008) | <0.001 |
| Model6 | 1.006 | (1.004-1.008) | <0.001 |

Hazard ratio and 95% CI for MHR in 28-day mortality were calculated using different Cox regression models. Model 1 adjusted for age and sex. Model 2 adjusted for model 1 plus lung infection, abdominal infection, urinary infection, and bloodstream infection. Model 3 adjusted for model 2 plus NEU, LYM, Hb and PLT. Model 4 adjusted for model 3 plus ALT, TB, and SCr. Model 5 adjusted for model 4 plus Lac, PaCO_2_ and Bicarbonate. Model 6 adjusted for model 5 plus LSBP, LDBP and NE.

**Table S3. Association between MHR group and 28-day mortality of sepsis patients in different subgroups.**

| 28-day mortality | | | | | | | | | |
| --- | --- | --- | --- | --- | --- | --- | --- | --- | --- |
|  | | | MHR<=92  Q1 | 92<MHR<=106  Q2 | | 106<MHR<=121  Q3 | | MHR>121  Q4 | |
| Subgroup | | N | Hazard ratio | Hazard ratio  95%CI | P value | Hazard ratio  95%CI | P value | Hazard ratio  95%CI | P value |
| TOTAL | | 11275 | Ref. | 1.049(0.926-1.187) | 0.455 | 1.265(1.118-1.431) | <0.001 | 1.369(1.211-1.549) | <0.001 |
| Sex | Male | 6581 | Ref. | 1.015(0.861-1.195) | 0.861 | 1.248(1.060-1.470) | 0.008 | 1.366(1.160-1.608) | <0.001 |
|  | Female | 4694 | Ref. | 1.096(0.905-1.328) | 0.346 | 1.284(1.063-1.549) | 0.009 | 1.368(1.132-1.652) | 0.001 |
| Age | >=70 | 4941 | Ref. | 1.058(0.903-1.240) | 0.485 | 1.236(1.053-1.452) | 0.01 | 1.328(1.130-1.557) | 0.001 |
|  | <70 | 6334 | Ref. | 1.023(0.835-1.253) | 0.828 | 1.233(1.012-1.501) | 0.037 | 1.296(1.063-1.581) | 0.010 |
| DM | Yes | 3435 | Ref. | 1.025(0.823-1.277) | 0.824 | 1.122(0.897-1.404) | 0.314 | 1.339(1.072-1.672) | 0.010 |
|  | No | 7840 | Ref. | 1.060(0.911-1.233) | 0.009 | 1.313(1.131-1.522) | <0.001 | 1.361(1.173-1.580) | <0.001 |
| HBP | Yes | 7270 | Ref. | 1.051(0.905-1.220) | 0.518 | 1.219(1.049-1.416) | 0.01 | 1.358(1.168-1.578) | <0.001 |
|  | No | 4005 | Ref. | 1.059(0.844-1.329) | 0.618 | 1.363(1.094-1.670) | 0.006 | 1.336(1.073-1.665) | 0.010 |
| CHD | Yes | 1350 | Ref. | 1.034(0.749-1.428) | 0.838 | 1.074(0.764-1.509) | 0.682 | 1.337(0.953-1.875) | 0.093 |
|  | No | 9925 | Ref. | 1.050(0.917-1.202) | 0.482 | 1.278(1.119-1.459) | <0.001 | 1.352(1.184-1.545) | <0.001 |
| CKD | Yes | 2440 | Ref. | 1.197(0.959-1.494) | 0.113 | 1.265(0.997-1.607) | 0.053 | 1.374(1.093-1.727) | 0.007 |
|  | No | 8835 | Ref. | 0.992(0.854-1.154) | 0.921 | 1.257(1.086-1.454) | 0.002 | 1.352(1.167-1.566) | <0.001 |
| APACHEIII | >=50 | 7022 | Ref. | 1.044(0.911-1.196) | 0.538 | 1.147(1.004-1.310) | 0.044 | 1.136(0.996-1.294) | 0.057 |
|  | <50 | 4253 | Ref. | 1.017(0.749-1.379) | 0.915 | 1.106(0.789-1.550) | 0.558 | 1.095(0.731-1.639) | 0.660 |
| NE | Yes | 4801 | Ref. | 1.030(0.878-1.207) | 0.718 | 1.241(1.063-1.449) | 0.006 | 1.228(1.052-1.434) | 0.009 |
|  | No | 6474 | Ref. | 1.050(0.859-1.282) | 0.636 | 1.226(0.999-1.504) | 0.051 | 1.498(1.220-1.839) | <0.001 |

Hazard ratios of 28-day mortality risk on the stratification of sex, age levels, coronary heart disease, diabetes, hypertension, chronic kidney disease and APACHE III. Adjusted variables included age, sex, lung infection, abdominal infection, urinary infection, bloodstream infection, NEU, LYM, Hb, PLT, ALT, TB, SCr, Lac, PaCO_2_, Bicarbonate, LSBP, LDBP and NE.

**
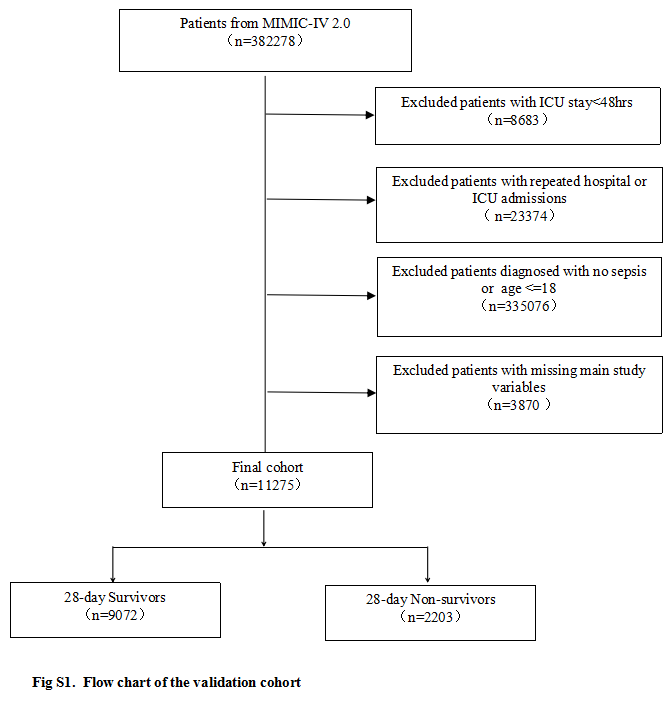
**

**
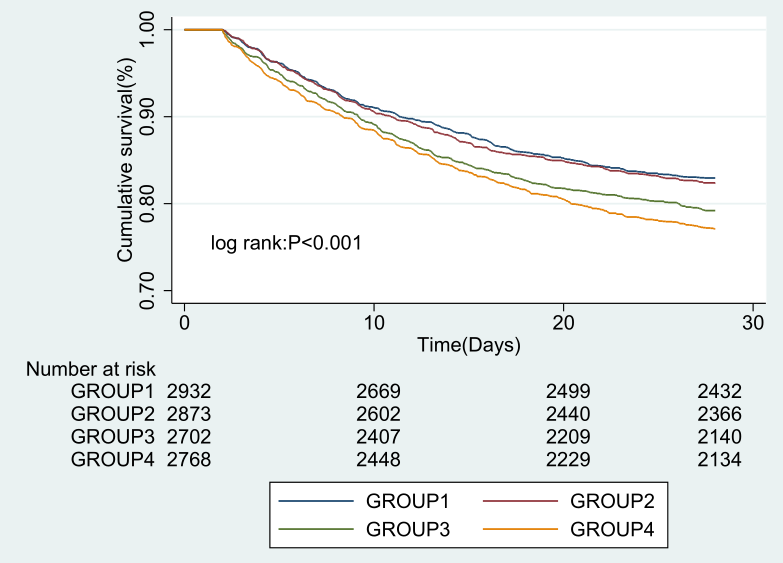
**

**Fig S2. Kaplan–Meier curves of 28-day mortality by MHR**

**
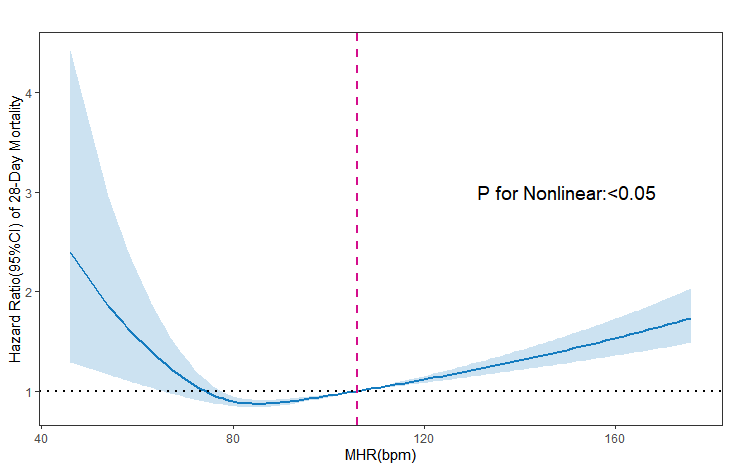
**

**Fig S3. Association between MHR and hazard ratio(95% CI) of 28-day mortality using a restricted cubic spline regression model.** Adjusted variables included age, sex, lung infection, abdominal infection, urinary infection, bloodstream infection, NEU, LYM, Hb, PLT, ALT, TB, SCr, Lac, PaCO_2_, Bicarbonate, LSBP, LDBP and NE. The reference (hazard ratio = 1, horizontal dotted line) was an MHR of 106 bpm (vertical dotted line). Solid lines indicate HRs, and shadow shape indicate 95% CIs. HRs hazard ratios, CI confidence interval.
